# Efficient Encapsulation and Controlled Release of the Pesticide Emamectin Benzoate in Polylactic Acid Microspheres Prepared by Modified Solvent Evaporation

**DOI:** 10.3390/molecules29246008

**Published:** 2024-12-20

**Authors:** Sheng Xu, Yamin Liu, Yilan Chen, Gang Wu

**Affiliations:** 1 School of Ecological Environment and Urban Construction, Fujian University of Technology, Fuzhou 350118, China; 19791028@fjut.edu.cn (S.X.); 19721651@fjut.edu.cn (Y.L.); 2 Key Laboratory of Biopesticide and Chemical Biology, Ministry of Education, Fujian Agriculture and Forestry University, Fuzhou 350002, China

**Keywords:** emamectin benzoate, polylactic acid, microspheres, modified solvent evaporation, controlled release

## Abstract

Emamectin benzoate (EB) is a highly effective broad-spectrum insecticide and acaricide. However, because EB is easily degraded, the conventional formulations of EB are often overapplied. In this study, polylactic acid (PLA)-based microspheres were prepared using the modified solvent evaporation method for the controlled release of EB. The microspheres were optimized to achieve higher EB loading. The effects of process parameters on the properties of microspheres, including encapsulation efficiency (EE), particle size, and pesticide loading, were investigated. Additionally, the controlled release behavior of EB microspheres was compared with that of conventional EB emulsifiable concentrate (EC). Spherical-shaped microspheres were obtained with an EE reaching 90.63 ± 1.90%, and introducing an external aqueous phase into the system can significantly enhance the EE of microspheres by over 30%. FTIR, DSC, and XRD analyses indicate that the preparation process of PLA microspheres was mainly physical encapsulation and had no chemical effect on EB. Notably, the EB microspheres displayed more potent control efficacy compared to commercial formulation EB EC against *Plutella xylostella*. The corrected mortality for the EB microspheres reached 90.00 ± 5.77% after 21 days of application, whereas the corrected mortality for the EB EC was only 19.23 ± 6.66% after 14 days of application. Our study demonstrates that EB-encapsulated PLA microspheres have strong potential as environmentally friendly control release EB formulations.

## 1. Introduction

Emamectin benzoate (EB) is a highly effective insecticide and acaricide, noted for its broad spectrum of activity, low toxicity, and minimal environmental impact. It has been extensively employed in agricultural pest management. However, due to the degradability of EB in natural environments, conventional formulations often necessitate application at doses that exceed recommended levels [1,2,3]. These problems could be solved by the controlled release technology based on microencapsulation. Compared to non-encapsulated commercial formulations, the microspheres as a controlled release carrier, demonstrating sustained-release properties, resistance to UV degradation, and storage stability while minimizing skin contact toxicity. By employing appropriate microencapsulation processes and suitable carrier materials, the controlled release of pesticides can be effectively achieved [3,4,5].

There are various microencapsulation technologies, including coacervation, spray drying, polymerization, and solvent volatilization, etc. The coacervation method has a high encapsulation efficiency, but it is costly and poses a risk of residual coagulants and solvents [6,7,8]. The spray drying method is simple to operate; however, it is not suitable for temperature-sensitive substances and requires an amount of energy [9,10]. The polymerization method exhibits a rapid reaction rate; however, the polymerization process is complicated, and there are monomer residues within the reaction system [11,12]. Among various microcapsule technologies, solvent evaporation technology is particularly favored due to its simple equipment requirements and easy operation. Nonetheless, this method necessitates careful selection of encapsulation conditions and carrier materials to achieve high encapsulation efficiency [3,4,5,13,14,15,16]. To enhance the EE of EB microspheres, we modified solvent evaporation to microencapsulate EB in this study.

Many different active materials like drugs, enzymes, vitamins, pesticides, flavors, and catalysts have been successfully encapsulated inside microspheres or microcapsules. These microspheres or microcapsules are made from a wide range of polymeric and non-polymeric materials, including polylactic acid, polyurea, polyethylene glycols, cellulose, gelatin, chitosan, cyclodextrins, etc. [3,4,5,6,7,8,9,10,11,12,13,14,15,16,17,18,19,20]. Biodegradable polylactic acid (PLA) is an FDA-approved polymer that has been extensively utilized as a drug carrier in the medical field [19]. Moreover, PLA can be synthesized from various bio-based resources, and its final metabolites are CO_2_ and H_2_O, both of which are non-toxic to human health and the environment. The commercially available EB formulations primarily consist of EC, water-dispersing granule (WDG), microemulsion formulation (ME), and granule formulation. In order to develop safe and environmentally friendly pesticide formulations, we used PLA as the encapsulation material to prepare EB-encapsulated microspheres. PLA has successfully encapsulated some types of pesticides, including combination of Spinosad and EB [3], azoxystrobin [15], cypermethrin [18], and lambda cyhalothrin [20]. These studies indicate PLA is an appropriate material for the encapsulation of pesticides. But the characteristics of the microspheres are significantly influenced by the microencapsulation method and the physicochemical properties of the active ingredients being encapsulated. We determined the optimal preparation conditions for EB-encapsulated PLA microspheres through single-factor studies on process parameters and modified the solvent evaporation method. The results indicated that the conventional oil–water volume ratio results in low EE of EB. By introducing an external water phase and significantly increasing the oil–water volume ratio, an EE exceeding 90% can be achieved. Compared with EB EC, we tested the control efficiency of EB-encapsulated PLA microspheres against sensitive second instar larvae of *P. xylostella*. The period of efficacy of EB microspheres (corrected mortality of 90.00 ± 5.77% on the 21st day after application) significantly surpassed that of EB EC (corrected mortality of 19.23 ± 6.66% on the 14th day, 0% on the 21st day after application). Therefore, the findings of this study can be effectively utilized in the research and application of environmentally friendly control release EB formulations.

## 2. Results and Discussion

### 2.1. Effect of Emulsifier Gelatin Concentration on the Microspheres Properties

**Figure 1:**
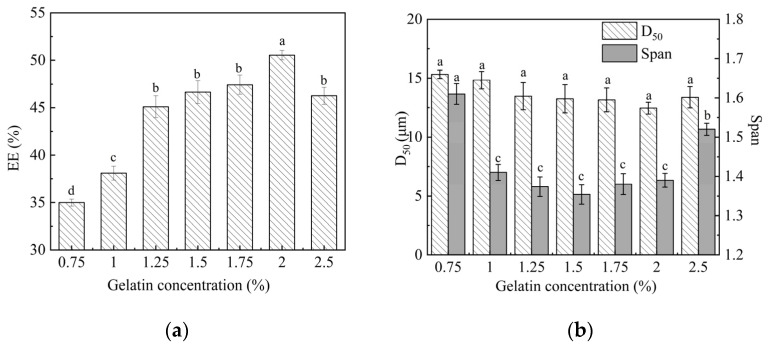
Effect of gelatin concentration on the EE (**a**) and particle size (**b**) of microspheres. Note: These microspheres were prepared without the external aqueous phase. Different lower-case letters indicate significant differences (*p* < 0.05).

Gelatin was selected as the emulsifier in the aqueous phase. Figure 1 shows the effect of gelatin concentration (0.75% to 2.5%) on EE and particle size of microspheres. As shown in Figure 1a, when the gelatin concentration increased from 0.75% to 2.0%, the EE of the microspheres gradually increased from 34.99 ± 0.36% to 50.54 ± 0.53%. Further increasing the gelatin concentration, the EE showed a decreasing trend. This phenomenon is mainly related to the role of emulsifiers in promoting the formation of interfacial film. During emulsification, the emulsifier is adsorbed on the surface of the dispersed droplets to promote film formation. A low concentration of emulsifiers cannot effectively promote film formation around microspheres, resulting in instability of the system and low EE. The film strength at the lotion interface increases with the increase in gelatin concentration, leading to a corresponding rise in EE of microspheres [15,21]. However, the addition of emulsifiers will increase the viscosity of the continuous phase, which can subsequently diminish the fluidity of the system. Consequently, when the gelatin concentration exceeds 2%, PLA may begin to precipitate prior to achieving uniform dispersion within the continuous phase, resulting in a decrease in EE of microspheres.

From Figure 1b, it can also be seen that the change in gelatin concentration had no significant effect on the D_50_ of microspheres, which remained basically unchanged at 12 μm. However, it had a significant impact on the span of particle sizes. The emulsifier can establish a spatial barrier at the oil–water interface, thereby preventing the droplets from approaching one another. When the gelatin concentration increased from 0.75% to 1.5%, the span of particle sizes decreased from 1.61 ± 0.03 to 1.35 ± 0.02. Subsequently, span increased with the increase in gelatin concentration. As previously analyzed, when the emulsifier concentration was higher, PLA began to precipitate before uniformly dispersing in a continuous phase, resulting in the uneven distribution of microsphere particle size.

The above analysis indicates that emulsifiers must maintain an appropriate concentration to ensure effective encapsulation and dispersion performance of microspheres. Therefore, the suitable concentration of gelatin is 1.5%.

### 2.2. Effect of an External Aqueous Phase and High Speed Shearing Time on the Microsphere Properties

**Figure 2:**
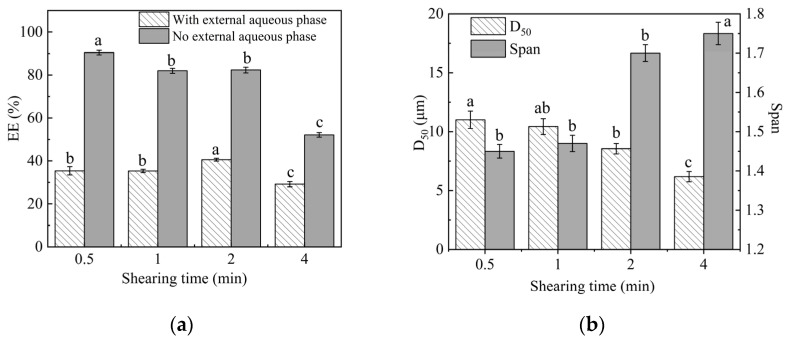
Effect of shearing time on the EE (**a**) and particle size (**b**) of microspheres. Note: Different lower-case letters indicate significant differences (*p* < 0.05).

The effect of the external aqueous phase and shearing time on EE and the particle size of microspheres are shown in Figure 2. Figure 2 shows that after adding external aqueous phase (V_oil phase_:V_internal aqueous phase_:V_external aqueous phase_ = 1:25:100), the EE of the microspheres significantly increased from 35.4 ± 2.68% to 90.44 ± 1.08%. The reason may be that the key of microsphere deposition lies in the initial stage of solidification. Adding a sufficient volume of water as the external aqueous phase in the initial stage of solidification helps the oil phase containing acetone diffuse from the initial emulsion to the water phase, thus enhancing the concentration gradient of PLA at the oil–water interface, promoting effective emulsification and EB encapsulation before solvent volatilization and thus improving the EE of the microsphere [16].

Figure 2 also shows that the EE decreased from 90.44 ± 1.08% to 52.15 ± 1.08% and that the span of particle sizes gradually increased to exceed 1.5 as the shearing time increased from 0.5 min to 4 min, following the addition of an external aqueous phase. Although high-speed shearing helps evaporate organic solvents, extending the shearing time can hinder the timely solidification and precipitation of the wall material PLA, which may cause the already formed sediment to rupture, ultimately leading to a decrease in EE and an increase in span.

As a result, the optimal shearing time is 0.5 min.

### 2.3. Effect of Oil–Water Volume Ratio on Microsphere Properties

**Figure 3:**
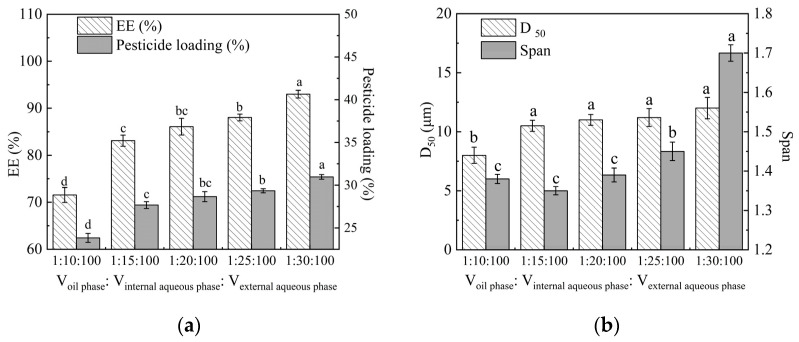
Effect of oil–water volume ratio on the EE (**a**) and particle size (**b**) of microspheres. Note: Different lower-case letters indicate significant differences (*p* < 0.05).

Figure 3 shows the effect of oil–water volume ratio on EE and the particle size of microspheres. When the ratio of V_oil phase_:V_internal aqueous phase_:V_external aqueous phase_ ranged from 1:10:100 to 1:30:100, the EE and pesticide loading (PL) exhibited an increasing trend. Notably, when the volume of the internal aqueous phase increased from ten times to fifteen times that of the oil phase, both EE and PL increased significantly. That is, EE increased from 71.55 ± 1.59% to 83.10 ± 1.19%, while PL increased from 23.85 ± 0.75% to 27.67 ± 0.40%. Subsequently, EE gradually increased to 93 ± 0.81%. Appropriately increasing the volume of the aqueous phase facilitated the complete dispersion of PLA and EB within the oil phase in the continuous phase, thereby enhancing their deposition and encapsulation at the oil–water interface.

Figure 3 also shows that the D_50_ rose from 8.0 ± 0.97 μm to 12.01 ± 1.29 μm, while the span significantly increased from 1.45 ± 0.02 to 1.7 ± 0.00 as the proportion of the internal aqueous phase increased. The presence of the external aqueous phase and the increase in the initial emulsion volume correspondingly weakened the shear and stirring intensity within the emulsion system. When the shear and stirring forces within the system were insufficient to maintain a uniform dispersion of microspheres, the span of particle sizes increased.

Therefore, the optimal oil-water volume ratio in the system is V_oil phase_:V_internal aqueous phase_:V_external aqueous phase_ at 1:25:100.

### 2.4. Effect of PLA Concentration on Microsphere Properties

**Figure 4:**
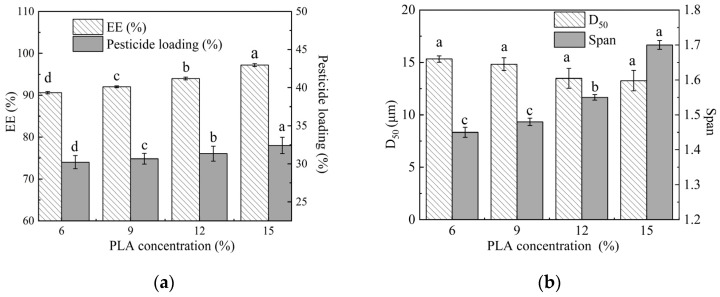
Effect of PLA concentration on the EE (**a**) and particle size (**b**) of microspheres. Note: Different lower-case letters indicate significant differences (*p* < 0.05).

The effect of the PLA concentration in the oil phase on EE and particle size of microspheres are shown in Figure 4. As the PLA concentration increased from 6% to 15%, the EE of the microspheres gradually rose from 90.63 ± 0.86% to 97.21 ± 1.07%. As the mass of PLA per unit volume increased, the possibility of contact between PLA and EB also increased. This heightened interaction accelerated the encapsulation process of PLA around EB, thereby enhancing the EE of the microspheres. In addition, as shown in Figure 4, it can be observed that when the concentration of PLA exceeded 9%, the span of particle sizes rose from 1.48 to 1.7. This indicates that the particle size distribution became obviously more uneven. PLA is a hydrophobic polymer, and therefore increasing the concentration of PLA in the organic phase leads to a relatively slow migration rate of the organic phase to the continuous phase. When the PLA concentration exceeds 9%, PLA is excessive, resulting in insufficient uniformity of the microspheres formed after solidification [16,22]. The optimal concentration of PLA in the oil phase is 6%.

### 2.5. Effect of Core–Wall Ratio on the Properties of Microspheres

**Figure 5:**
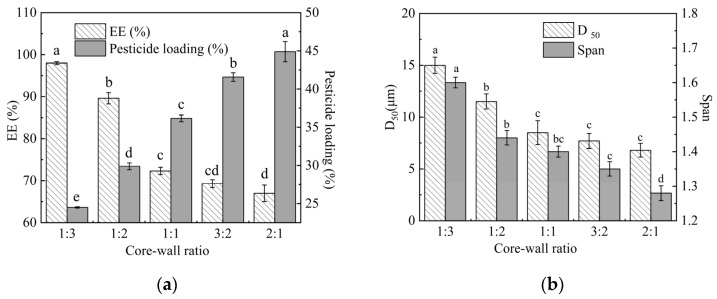
Effect of core–wall ratio on the EE (**a**) and particle size (**b**) of microspheres. Note: Different lower-case letters indicate significant differences (*p* < 0.05).

The effect of the core–wall ratio on the EE and particle size of microspheres is shown in Figure 5. As the core–wall ratio increased from 1:3 to 3:2, the pesticide loading of microspheres increased from 24.5 ± 0.09% to 44.89 ± 1.32%, while EE decreased from 97.98% ± 0.50% to 67% ± 1.98%. When the theoretical pesticide load exceeded 50%, specifically at a core–wall ratio of 1:1, there was a significant decline in the actual EE of microspheres. At a core–wall ratio of 2:1, EE dropped to 67 ± 1.98%, indicating that approximately one-third of EB was wasted. As the proportion of EB within the system increased, there was insufficient PLA mass to adequately encapsulate EB. Consequently, EB within the microsphere can easily detach from it, which leads to a decrease in EE and D_50_. To ensure optimal EE of microspheres and appropriate effective content of EB within microspheres, the most suitable core-wall mass ratio is 1:2.

### 2.6. The Character of an EB-Encapsulated PLA Microsphere

According to the optimal experimental conditions established in previous studies, control release microspheres with a high encapsulation efficiency were prepared. Their characteristics were characterized and analyzed.

#### 2.6.1. SEM Analysis

**Figure 6:**
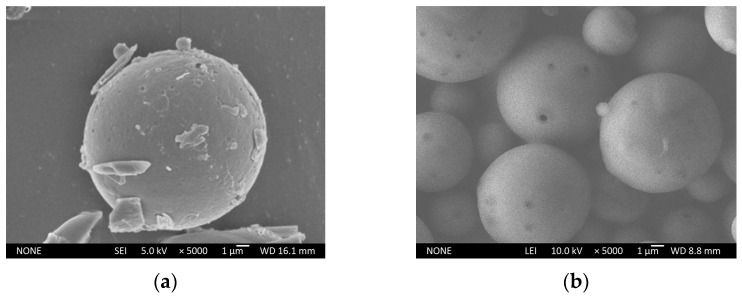
SEM of microspheres fabricated by different preparation methods (×5000): (**a**) conventional solvent evaporation; (**b**) modified solvent evaporation.

Figure 6 illustrates that PLA microspheres are spherical, with no observable adhesion between the microspheres. However, from Figure 6a, the particle size of the microspheres prepared by conventional solvent evaporation exceeded 15 μm, and there was a notable accumulation of debris on their surfaces. This phenomenon can be attributed to the limited volume of the continuous water phase, which hinders effective dispersion of the microspheres and leads to wastage of both core and wall materials. The findings align with the observation that the EE of microspheres was below 40%. From Figure 6b, it can be seen that the microspheres prepared by modified solvent evaporation demonstrated a smooth surface free from debris adhesion and possessed significantly smaller particle sizes compared to those produced by the conventional method.

#### 2.6.2. FTIR Analysis

**Figure 7:**
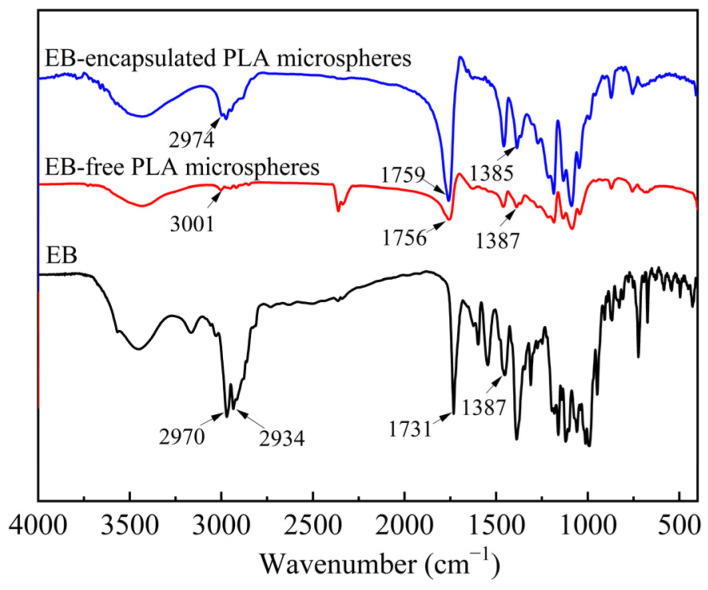
FTIR spectra of EB, EB-free PLA microspheres, and EB-encapsulated PLA microspheres.

Figure 7 illustrates the FTIR spectra of EB, EB-free PLA microspheres, and EB-encapsulated PLA microspheres. In comparison to the FT-IR spectra of both EB and EB-free PLA microspheres, the FTIR spectrum of the EB-encapsulated PLA microspheres did not exhibit a disappearance of corresponding characteristic peaks or the emergence of new peaks; however, there was a significant alteration in the intensity of these characteristic peaks. As depicted in Figure 7, the characteristic peaks at 2974 cm^−1^ (-CH_3_ stretching vibration), at 1759 cm^−1^ (C=O stretching vibrations), and at 1385 cm^−1^ (-CH_3_ symmetric bending vibration) were present in the FTIR spectra of all samples. The intensities of these characteristic peaks in the spectrum for EB-encapsulated PLA microspheres fell between those observed for both EB and EB-free PLA microsphere samples. This variation can be attributed to alterations in the proportions of EB and PLA within the sample following the encapsulation of EB by PLA. These findings indicate that EB was successfully encapsulated within PLA microspheres without any substantial chemical alterations occurring within the functional groups of EB.

#### 2.6.3. DSC Analysis

**Figure 8:**
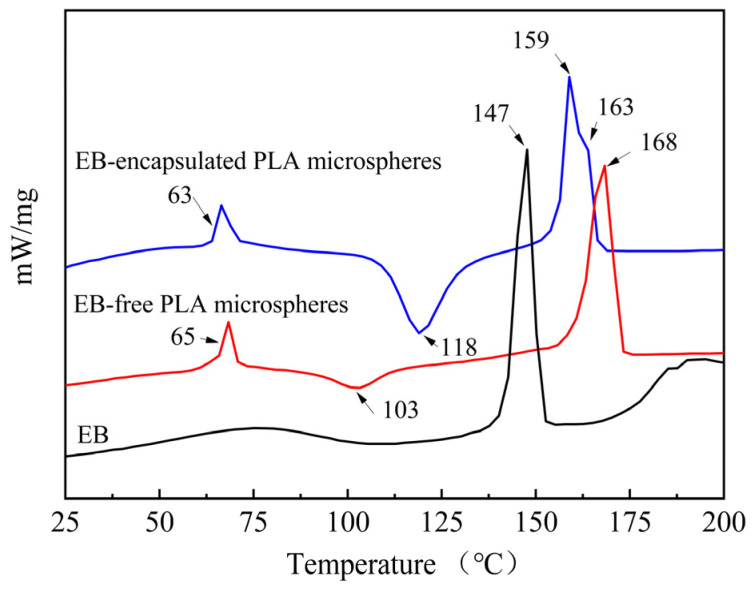
DSC analysis of EB, EB-free PLA microspheres, and EB-encapsulated PLA microspheres.

The compatibility between EB and PLA within microspheres was analyzed by DSC. Figure 8 shows the DSC curves of EB, EB-free PLA microspheres, and EB-encapsulated PLA microspheres. For EB, there was no glass transition temperature (T_g_) observed, and a broad endothermic peak appeared at 77.68 °C, attributed to the loss of adsorbed water from EB. Additionally, the melting peak (T_m_) of EB was around 147 °C. With increasing temperature from 25 °C to 200 °C, the DSC curves of both PLA and EB-encapsulated PLA microspheres possessed three thermal characteristics: T_g_, T_m,_ and a cold crystallization peak (T_c_). The T_g_ of PLA was about 65 °C, while the T_g_ of EB-encapsulated PLA microspheres was about 63 °C. The T_c_ of PLA was at 103 °C; however, the T_c_ of EB-encapsulated PLA microspheres was observed to be at 118 °C. Compared to the T_g_ and T_c_ of PLA, the observed shifts in T_g_ and T_c_ for the EB-encapsulated PLA microspheres clearly indicated the impact of the encapsulated EB. It is worth noting that PLA had a single melting peak at about 168 °C, while the EB-encapsulated PLA microspheres showed double melting peaks that were around 159 °C and 163 °C, respectively. The melting peaks of EB-encapsulated PLA microspheres were between the melting temperatures of EB and PLA. Compared to thermal characteristics of EB and PLA, the observed changes in the three thermal characteristics of the EB-encapsulated PLA microspheres can be attributed to the encapsulation of EB within PLA. The results presented above confirm that during preparation by modified solvent evaporation, PLA and EB were not simply physically mixed; rather, they demonstrated a certain degree of compatibility, as supported by the findings from FTIR analysis.

#### 2.6.4. XRD Analysis

**Figure 9:**
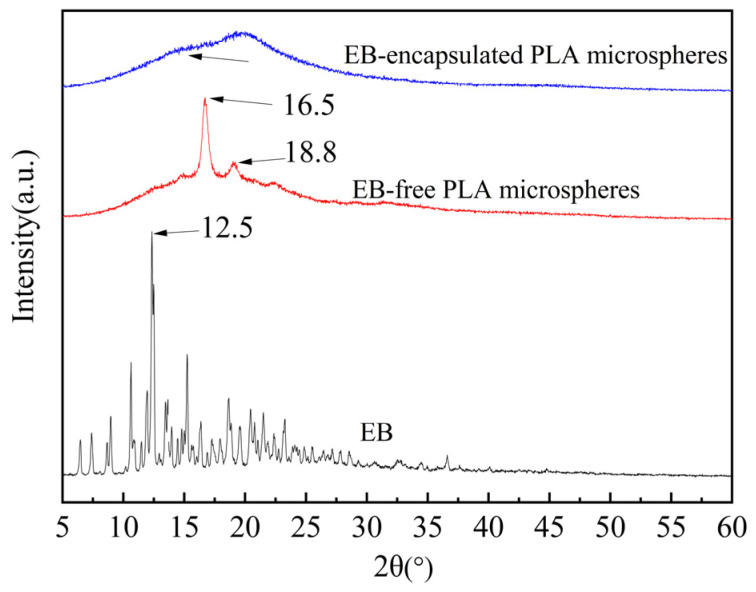
XRD patterns of EB, EB-free PLA microspheres, and EB-encapsulated PLA microspheres.

The XRD diffraction patterns of EB, EB-free PLA microspheres, and EB-encapsulated PLA microspheres are presented in Figure 9. The spectra for EB exhibited a series of sharp diffraction peaks, with a particularly strong peak at about 2θ = 12.5°. And the spectrum for PLA had two distinct sharp diffraction peaks at about 2θ = 16.5° and 18.8°. However, these sharp peaks of EB and PLA were unable to be found from the XRD patterns of EB-encapsulated PLA microspheres, which were replaced by a wide and diffuse peak. This observation suggests that the crystalline state of the microspheres system was altered, which may be attributed to encapsulation of EB within the wall material PLA.

### 2.7. The Indoor Virulence of EB-Encapsulated PLA Microspheres Against P. xylostella

Table 1 presents the results of indoor virulence tests performed on a 3% EB microsphere suspension against sensitive second instar larvae of *P. xylostella*. The LC_50_ of the EB microsphere suspension was 0.0028 mg/L, measured 48 h after application, while the LC_50_ of the EB EC was 0.00025 mg/L. The results indicate that the samples utilized in this study were sensitive to EB, and that the EB formulations demonstrated insecticidal activity. However, the LC_50_ of the EB microsphere suspension was higher than that of the EB EC, suggesting that the EB-encapsulated PLA microspheres possessed a certain degree of sustained release performance.

### 2.8. Control Efficacy of EB-Encapsulated PLA Microspheres Against P. xylostella

Table 2 shows the results of the control efficacy of EB-encapsulated PLA microspheres and EB EC against the sensitive second instar larvae of *P. xylostella*. It indicates that the control efficacy of EB microsphere suspension exceeded that of EB EC. Notably, the corrected mortality of the EB microsphere suspension was maintained over 90% for up to 21 days after application. In contrast, the corrected mortality of the EB EC significantly declined to approximately 50% on the 7th day, and EB EC demonstrated no insecticidal activity on the 21st day. These findings suggest that the sustained efficiency of EB-encapsulated PLA microspheres effectively prolongs the lasting validity period of EB after microencapsulation treatment.

## 3. Materials and Methods

### 3.1. Materials

The chemical agents utilized in this study were as follows: technical grade EB with a purity of 95%, sourced from Hubei Jinghong Biotech Co., Ltd., Xiangyang, China; PLA of technical grade (M_w_ = 80,000), obtained from Shenzhen Guanghua Weiye Industrial Co., Ltd., Shenzhen, China; and gelatin supplied by Sinopharm Chemical Reagent Co., Ltd., Shanghai, China. The methanol employed for HPLC was of chromatographic grade.

### 3.2. Preparation of EB-Encapsulated PLA Microspheres

Total amounts of 0.1895 g of EB and 0.36 g of PLA were dissolved in 6 mL of a mixed organic solvent comprising dichloromethane (4 mL) and acetone (2 mL), resulting in a PLA concentration of 6%. This organic solution (6 mL) was used as the oil phase. The 1.5% gelatin solution (150 mL) was subsequently prepared to serve as the internal aqueous phase. The oil phase was then promptly added to the internal aqueous phase, and the system was emulsified through high-speed 7000 rpm for 0.5 min, utilizing a high-speed disperser to create an O/W initial emulsion. Then, using distilled water (600 mL) as the external aqueous phase, the O/W initial emulsion was mixed and stirred with the external aqueous phase at a speed of 700 rpm for 4 h under dark conditions. Following filtration, washing, centrifugation, and vacuum drying at a temperature of 36 °C, EB-encapsulated microspheres were successfully prepared.

A single-factor exploration method was used to study the effects of different factors (including gelatin concentration, shearing time, oil–water volume ratio, PLA concentration, and core–wall ratio) on the encapsulation efficiency (EE), D_50_, and particle size distribution of microspheres.

### 3.3. Measurement of EB Loading Rate and Encapsulation Efficiency of Microspheres

The EB microspheres were dissolved in dichloromethane. Following the complete volatilization of dichloromethane, the extracted EB from the microspheres was subsequently dissolved in methanol. The EB concentration in the methanol was quantified using HPLC (UltiMate 3000, Dionex, Sunnyvale, CA, USA) with a UV detector.

The column employed for HPLC analysis was an Amemyst C_18_ (250 mm × 4.6 mm). The mobile phase consisted of a mixture of methanol and triethylamine solution in a ratio of 95:5 (*v*/*v*). The triethylamine solution was prepared by diluting triethylamine in water at a ratio of 1:1000 (*v/v*). The flow rate was set to 1.0 mL/min, with the column temperature maintained at 25 °C, and the detection wavelength was established at 245 nm. An injection volume of 20 µL was utilized for each sample.

Pesticide loading is defined as the weight percentage of the actual encapsulated amount of EB in relation to the total weight of the microspheres. EE refers to the weight percentage of the actual encapsulated EB amount compared to initial EB quantity introduced into the system.

### 3.4. Measurement of Microsphere Particle Size Distribution

We dissolved 0.05 g of EB microspheres in 100 mL of a 1% K_2_HPO_4_ solution using ultrasound for 20 min. The dispersed sample was then injected into a laser particle size analyzer (MS3000, Malvern, Worcestershire, UK) for measurement. The particle size distribution was expressed by a span value, which was calculated using the following Equation (1):

(1) $$\mathrm{Span}=\frac{D_{90}-D_{10}}{D_{50}}$$

where

D_90_—90% of the particle sizes of microspheres are smaller than this value (μm);

D_10_—10% of the particle sizes of microspheres are smaller than this value (μm);

D_50_—50% of the particle sizes of microspheres are smaller than this value (μm).

### 3.5. Characterization of Microspheres

The morphology of EB microspheres was analyzed using scanning electron microscopy (SEM, JSM−5310LV, JEOL, Tokyo, Japan).

The chemical structure of the microspheres was analyzed using a Fourier transform infrared spectrometer (FTIR, Nicolet iS50, Thermo Fischer Scientific, Waltham, MA, USA).

The compatibility analyses of the samples were performed using differential scanning calorimetry (DSC214, NETZSCH, Selb, Germany). The nitrogen flow rate was maintained at 20 mL/min, while the heating rate was set to 10 °C/min.

X-ray diffraction patterns were performed using a powder X-ray diffractometer (XRD, D8 ADVANCE, Bruker, Mannheim, Germany), whose tube voltage was 40 KV and tube current was 40 mA with Cu Kα radiation (λ = 0.15406 nm).

### 3.6. Insect Bioassay

#### 3.6.1. The Indoor Virulence Tests of Microspheres

The virulence of EB microsphere suspension (active ingredient EB concentration of 3%) was assessed through the leaf dipping method against the sensitive second instar larvae of *P. xylostella* [23,24,25]. The EB microsphere suspension agent was prepared into seven series of concentration solutions using serial dilution method with a 0.1% Tween-80 aqueous solution. A 1.5% EB EC was utilized as the product control and diluted with the same method. A 0.1% Tween-80 solution served as the blank control. Each treatment was repeated four times. Mortality was recorded at 48 h post-leaf disc exposure, and the corrected mortality rate for each treatment was subsequently calculated using the following Equation (2):

Corrected mortality (%) = (mortality rate of the treatment group − mortality rate of the blank control group)/(1 − mortality rate of the blank control group) × 100(2)

#### 3.6.2. Control Efficacy of EB Microspheres

The EB microsphere suspension was diluted to achieve an EB solution at concentrations of 8 mg/L. The diluted solution was uniformly applied to the foliar of cabbage at a dosage of 60 mL/pot. A 1.5% EB EC was diluted to the same concentration, employed as the product control. Each treatment was repeated three times. The pot experiments were conducted under natural light conditions [26]. On the 1st day, 3rd day, 7th day, 14th day, and 21st day, the cabbage leaves measuring 6 cm × 6 cm were excised and subsequently placed in culture dishes. Ten sensitive second instar larvae of *P. xylostella* were introduced for feeding in each dish and maintained under controlled conditions (25 ± 1 °C, 60% relative humidity, a 16:8 (L:D) h photoperiod). Mortality was recorded at 48 h post-leaf disc exposure, and distilled water served as the blank control.

### 3.7. Statistical Analysis

Data were analyzed using one-way ANOVA followed by the DMRT test. Different lowercase letters were denoted to mark significant differences (*p* < 0.05). First, all averages were arranged in descending order, with the largest average designated as “a”. This average was then compared to subsequent averages; those that did not show a significant difference were also marked as “a” until an average exhibiting a significant difference was marked as “b”. The maximum average labeled “b” was subsequently established as the standard. Following this, unmarked averages were compared against it, with all non-significant differences continuing to be marked as “b” until a significantly different average appeared, which was marked as “c”. We repeated the above steps until the smallest average had a marked letter.

## 4. Conclusions

The present study reports an EB-encapsulated PLA microspheres prepared using a modified solvent evaporation method. The optimal reaction conditions were obtained: 6% PLA in oil phase, 1.5% gelatin in internal aqueous phase, shearing speed of 7000 rpm for 0.5 min, V_oil Phase_:V_internal aqueous phase_:V_external aqueous phase_ = 1:25:100, core–wall ratio = 1:2. Under these reaction conditions, EB microspheres with narrow particle size distribution and smooth surface were successfully prepared, whose EE was 90.63 ± 1.90%. Notably, compared to the conventional solvent evaporation method, incorporating an external aqueous phase into the system significantly enhanced the EE of microspheres by over 30%. Moreover, it can be verified that EB and PLA had certain compatibility within EB-encapsulated PLA microspheres by XRD and DSC. The FTIR analysis indicates that the process primarily involves physical encapsulation and does not have a chemical effect on EB.

The indoor virulence and control efficiency of EB-encapsulated PLA microspheres against the sensitive second instar larvae of *P. xylostella* were also evaluated and compared with EB EC. The results show that EB microspheres had good control release efficacy. The period of EB microsphere efficacy significantly surpassed that of EB EC. After application, the corrected mortality of EB microspheres was found to be 90.00 ± 5.77% on the 21st day. In contrast, the corrected mortality of EB EC was only 19.23 ± 6.66% on the 14th day, and EB EC had no insecticidal activity on the 21st day. The encapsulation of EB within PLA microspheres significantly extended EB’s lasting validity period.

In summary, EB-encapsulated PLA microspheres demonstrated a high loading capacity for EB and effective controlled release properties. These characteristics suggest it has good potential for agricultural application as an environmentally friendly control release EB formulation.

## Figures and Tables

**Table 1:** Toxicity of EB formulations against the second instar larvae of *P. xylostella*.

EB Formulation	Virulence Regression Equation Y = ax + b	LC_50_ (mg/L)	LC_50_ with a 95% Confidence Interval (mg/L)	Correlation Coefficient r
EB-encapsulated microspheres	y = 0.3283x + 0.8383	0.0028	0.0022~0.0035	0.9747
EB EC	y = 0.6798x + 2.4485	0.00025	0.00021~0.00030	0.9904

**Table 2:** The control efficacy of EB formulations against the second instar larvae of *P. xylostella* (mean ± S.E.).

EB Formulation	Corrected Mortality (%) After Application				
	1st day	3rd day	7th day	14th day	21st day
EB-encapsulated microspheres	85.19 ± 3.70 a	85.19 ± 3.70 b	93.33 ± 0.00 a	92.86 ± 3.57 a	90.00 ± 5.77 a
EB EC	93.33 ± 3.33 a	100.00 ± 0.00 a	50.00 ± 3.57 b	19.23 ± 6.66 b	0 b

Note: Different lower-case letters indicate significant differences (*p* < 0.05).

## Data Availability

Data will be made available on request.
